# Metabolic fingerprinting of bacteria by fluorescence lifetime imaging microscopy

**DOI:** 10.1038/s41598-017-04032-w

**Published:** 2017-06-16

**Authors:** Arunima Bhattacharjee, Rupsa Datta, Enrico Gratton, Allon I. Hochbaum

**Affiliations:** 1 Department of Chemical Engineering and Materials Science, University of California, Irvine, Irvine, CA 92697 USA; 2 Laboratory for Fluorescence Dynamics, Department of Biomedical Engineering, University of California, Irvine, Irvine, CA 92697 USA; 3 Department of Chemistry, University of California, Irvine, Irvine, CA 92697 USA

## Abstract

Bacterial populations exhibit a range of metabolic states influenced by their environment, intra- and interspecies interactions. The identification of bacterial metabolic states and transitions between them in their native environment promises to elucidate community behavior and stochastic processes, such as antibiotic resistance acquisition. In this work, we employ two-photon fluorescence lifetime imaging microscopy (FLIM) to create a metabolic fingerprint of individual bacteria and populations. FLIM of autofluorescent reduced nicotinamide adenine dinucleotide (phosphate), NAD(P)H, has been previously exploited for label-free metabolic imaging of mammalian cells. However, NAD(P)H FLIM has not been established as a metabolic proxy in bacteria. Applying the phasor approach, we create FLIM-phasor maps of *Escherichia coli*, *Salmonella enterica* serovar Typhimurium*, Pseudomonas aeruginosa, Bacillus subtilis*, and *Staphylococcus epidermidis* at the single cell and population levels. The bacterial phasor is sensitive to environmental conditions such as antibiotic exposure and growth phase, suggesting that observed shifts in the phasor are representative of metabolic changes within the cells. The FLIM-phasor approach represents a powerful, non-invasive imaging technique to study bacterial metabolism *in situ* and could provide unique insights into bacterial community behavior, pathology and antibiotic resistance with sub-cellular resolution.

## Introduction

Bacteria are found in diverse environments, and their ability to modulate metabolic processes in response to adverse conditions gives them unique survival advantages. In the natural environment, multiple species interact in complex niches and exhibit a range of community behavior^1–4^. In the medical environment, these metabolic changes make bacterial infections difficult to eliminate. Treatment of bacterial infections are often hindered by emergence of multi-drug resistant phenotypes^5, 6^. A major source of antibiotic resistance and tolerance are slow growing ‘persister’ phenotypes, which are often associated with biofilm formation and chronic infections^7–9^. Mapping the metabolic activity of bacteria within natural communities and medical infections, therefore, can provide insights into the role of metabolism in determining bacterial community behavior.

Fluorescence spectroscopy is a non-invasive technique which has been extensively used for metabolic imaging in mammalian cells. Fluorescence lifetime imaging microscopy (FLIM), in particular, is a powerful label-free method to probe the local environment and molecular conformation of endogenous fluorophores^10^. The FLIM signature of an autofluorescent metabolic coenzyme, reduced nicotinamide adenine dinucleotide (phosphate) (NAD(P)H), has been employed as an endogenous biomarker for metabolic activity of mammalian cells in cancer biology and the detection of stem cell differentiation^11–17^. NAD(P)H in its free state has a significantly shorter fluorescence lifetime (0.4 ns), due to self-quenching, than its protein bound state and is thus easily discernable by FLIM^18^. FLIM measurements allow mapping of NAD(P)H lifetimes with sub-cellular resolution. The relationship between the NAD(P)H fluorescence lifetime and metabolic activity, however, has not been established in bacteria.

As in the case of mammalian cells, bacteria produce many endogenous fluorescent molecules with distinct spectral characteristics^19, 20^, some unique to specific bacteria^21, 22^, rendering them as promising probes for identification and characterization. Fluorescence spectroscopy of some intrinsic fluorophores has been previously explored in bacteria for the detection, differentiation, and characterization of various species^23–27^. The relationship between cytosolic concentrations of NAD(P)H in bacteria and their metabolic activity has been previously investigated^28–32^, and differences in the ratio of NAD^+^ and NADH are correlated with different metabolic states under aerobic and anaerobic conditions, dissolved oxygen tension, and growth phase^33–35^. Fluorescence lifetime measurements of bacteria have been previously reported in attempts to detect and differentiate bacterial species^34, 36, 37^. More recently, NAD(P)H fluorescence lifetime measurements were obtained from bacteria infecting mammalian cells, though no relationship to metabolic activity was determined^38^.

A previously established phasor approach to FLIM is a fit-free technique which does not require *a priori* knowledge of the fluorescent species in the sample^39, 40^. In brief, lifetime decay information from each pixel of the image is analyzed via Fourier transform to obtain the corresponding phasor position and the resulting 2-D histogram is plotted as a distribution on the phasor plot with coordinates (*g*,*s*). The *g* coordinate of the phasor plot extends between 0 and 1 while the *s* coordinate has values between 0 and 0.5. All single exponential lifetime decays fall on the ‘universal circle’ defined as a semi-circle of radius 0.5 between points (0,0) and (1,0) on the phasor plot. Phasors corresponding to a fluorescence lifetime value of 0 will fall on point (1,0) while longer lifetime signals will shift towards point (0,0) with increasing lifetime. The phasor of a mixture of two molecular species (each with single exponential lifetimes) will lie inside the universal circle on the line joining the phasor position of the two pure species, depending on the fractional contribution of each component. Hence, the position of the FLIM-phasor is directly related to the ratio of free to enzyme-bound NAD(P)H^41, 42^. A ‘right shift’ of the phasor indicates a larger free to bound ratio while a decrease in the ratio is indicated by a ‘left shift’ to coordinates corresponding to longer lifetimes. Lifetimes within the phasor distribution from a sample can be mapped back onto the acquired image to form the corresponding FLIM map. NAD(P)H FLIM-phasor has been successfully applied to study metabolism and oxidative stress in tumors^16, 17, 43^, cardiomyocytes^16^, metabolic fingerprinting of macrophages^44^, stem cell differentiation^15^, and in *Lactobacillus acidophilus* cultures^45^.

In this work, we demonstrate the potential of the NAD(P)H FLIM-phasor technique to differentiate metabolic states in live bacterial populations at the single-cell level. Phasor fingerprints were generated for immobilized planktonic cell populations of *Escherichia coli*, *Salmonella enterica* serovar Typhimurium*, Pseudomonas aeruginosa, Bacillus subtilis*, and *Staphylococcus epidermidis*. The phasor positions of the cells, corresponding to the free to bound NAD(P)H ratio, was found to depend on species, growth phase, and history of exposure to antibiotics and nutrient media. The phasor distribution shifted to shorter (free) NAD(P)H fluorescence lifetimes with exposure to antibiotics, and recovered to longer (bound) lifetimes when bacteriostatic antibiotics were administered and washed away. Moreover, the diffraction-limited FLIM maps achieved here display differences in the spread of phasor positions of individual cells within the same population under different environmental treatments. As a function of growth phase, the NAD(P)H lifetime phasor distribution changed non-monotonically. This suggests that the FLIM-phasor position captures information about metabolic activity not simply inferred by cell density measurements. These results demonstrate that FLIM of NAD(P)H in live bacterial cultures can be directly related to changes in metabolic activity, and that this method can provide distinct and nuanced information about metabolic states of bacteria *in situ* as compared to existing fluorescence spectroscopy and metabolomic techniques.

## Results

### Fluorescence lifetime phasor fingerprint of different bacterial species

**Figure 1:**
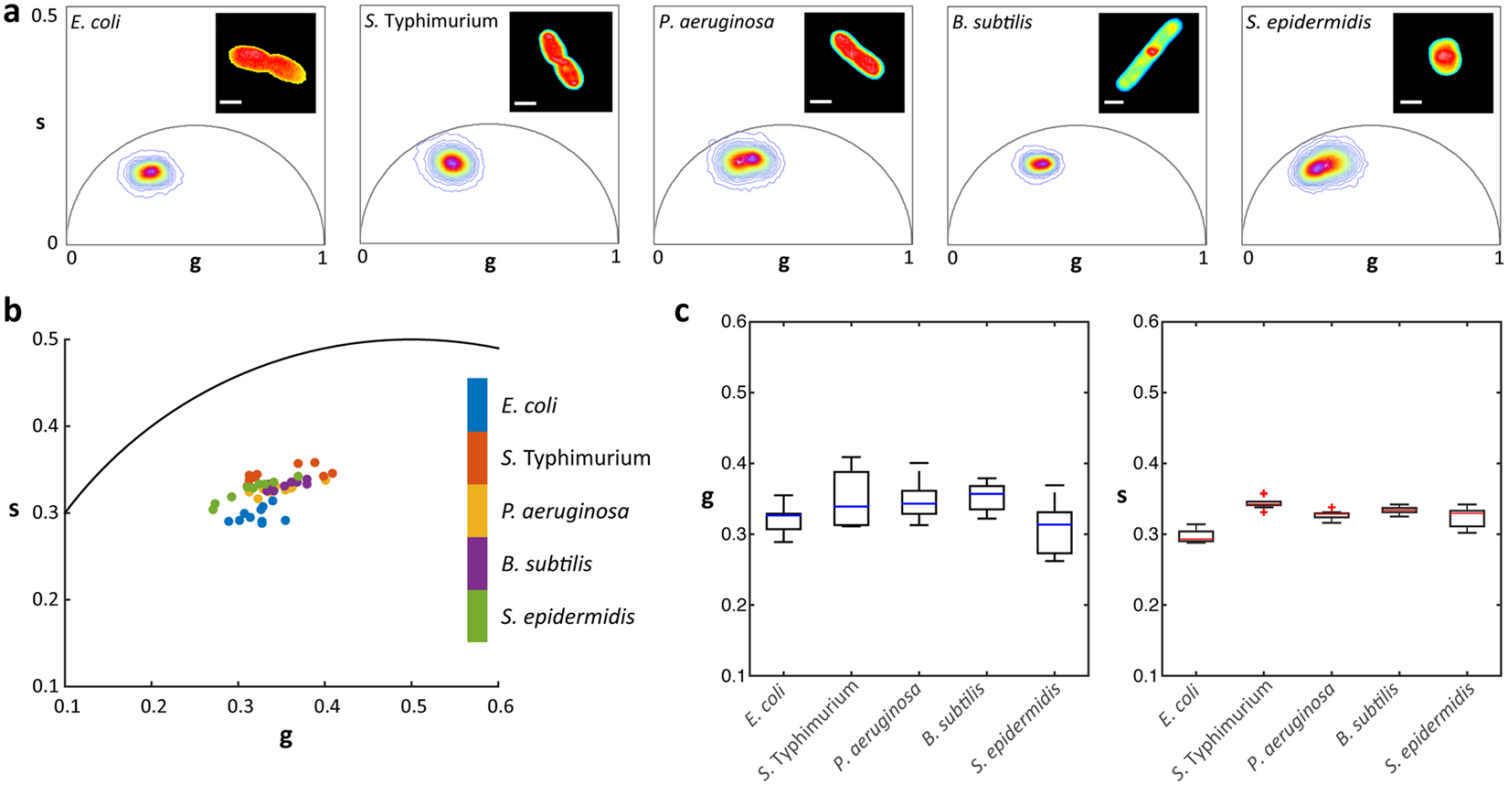
Bacterial phasor fingerprints. (**a**) Phasor distribution of *E. coli, S*. Typhimurium, *P. aeruginosa*, *B. subtilis* and *S. epidermidis*. Inset panels are representative fluorescence intensity images of each bacterium. (**b**) Bacterial phasor fingerprints of individual cells within each bacterial population. (**c**) Distribution of *g* and *s* within each bacterial population. Scale bars are 1 µm.

Two-photon FLIM of five clinically relevant species of bacteria, *E. coli, S*. Typhimurium*, P. aeruginosa, B. subtilis and S. epidermidis,* was performed on planktonic cells immobilized in agarose. Each species was imaged at a mid-exponential phase of growth to generate a phasor fingerprint (Fig. 1a and Supplementary S1). A comparative analysis of all the phasor coordinates of each bacterial cell, represented by a single data point, within each species population (*n* = *10*) shows variations along both *g* and *s* axes of the phasor plot (Fig. 1b). The bacterial phasor distribution displays greater variation along the *g* axis (between 0.26 to 0.41) than the *s* axis on the phasor plot (Fig. 1c). Supplementary Fig. S2 shows the fluorescence emission spectra of the five bacterial species (*n* = *10*) excited at 740 nm.

### Response of *E. coli* phasor to antibiotic exposure

**Figure 2:**
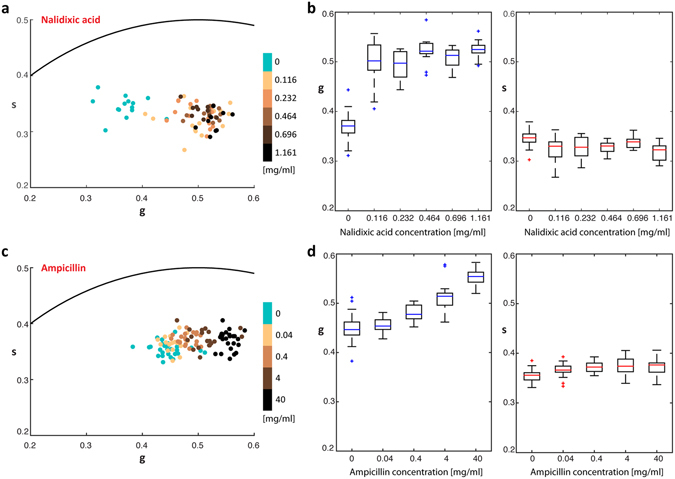
Bacterial phasor response to bacteriostatic and bactericidal antibiotic treatment. (**a**) Bacterial phasor of *E. coli* control culture (cyan) and culture treated with increasing concentrations of a bacteriostatic antibiotic, nalidixic acid (light to dark brown). (**b**) Distribution of *g* and *s* within populations of cells at each concentration of nalidixic acid. (**c**) Bacterial phasor of *E. coli* control culture (cyan) and culture treated with increasing concentrations of bactericidal antibiotic, ampicillin (light to dark brown). (**d**) Distribution of *g* and *s* within populations of cells at each concentration of ampicillin.

Bacteria encounter various stresses in their natural environments which elicit specific and highly regulated responses via changes in their metabolism^7, 9, 46, 47^. To determine the relationship between the FLIM phasor and metabolic activity, we probed the phasor response of *E. coli* to antibiotic treatment. Agarose embedded cells were treated with nalidixic acid or ampicillin, a bacteriostatic antibiotic and bactericidal antibiotic, respectively, then NAD(P)H FLIM was performed. The phasor position of *E. coli* cells treated with nalidixic acid shifted towards longer fluorescence lifetimes with increasing concentration (light to darker shades of brown dots), as compared to the untreated control population (cyan dots) (Fig. 2a). The *g* distribution exhibited a sharply increasing mean value at the lowest nalidixic acid concentration, while the mean *s* values were weakly sensitive to nalidixic acid concentration (Fig. 2b). *E. coli* cells similarly exposed to increasing concentrations of a bactericidal antibiotic, ampicillin, also exhibited corresponding phasor position shifts to larger *g* values, while *s* values remained relatively constant with ampicillin concentration (Fig. 2c and d) when compared with cells in control conditions. These shifts of the phasor position of cells treated with antibiotics corresponds to a concentration-dependent increase in the fraction of dead cells when stained in a live/dead assay (Supplementary Figs S3 and S4). Exposure of *E. coli* cells to the same volume of water for the same amount of time did not cause a shift and the position of the phasor was maintained at the same position as the control population (Supplementary Fig. S9).

To benchmark the FLIM phasor data with an independent metabolic activity measure, we used a resazurin assay to quantify aerobic respiration in planktonic cultures. Resazurin is a redox indicator, sensitive to the presence of oxygen and reducing equivalents, such as NADH. Under reducing conditions, resazurin is reduced to the fluorescent compound, resorufin, and the fluorescence intensity has been linked to bacterial respiration rates^48, 49^. Exposure of *E. coli* to the bacteriostatic nalidixic acid resulted in a significant drop in culture respiration, even at the lowest concentration tested in our study (Supplementary Fig. S5). Exposure of *E. coli* to bactericidal ampicillin, however, resulted in a slight increase in metabolic activity for cells exposed to 0.04 mg/mL ampicillin, which decreased with further increases in ampicillin concentration. The resazurin data are consistent with results and trends in the FLIM phasor data, which show a large shift to higher free to bound NAD(P)H ratios with exposure to 0.116 mg/mL nalidixic acid, and little to no concentration dependence thereafter (Fig. 2b). The FLIM data do not show a shift to lower free to bound NAD(P)H at 0.04 mg/mL ampicillin, but the data is within error of the starting culture and the change indicated by the resazurin assay is small relative to that observed for nalidixic acid exposure (Fig. 2d). The resazurin assay and FLIM data, however, show a trend to a lower respiration rate and higher free to bound NAD(P)H ratio, respectively, with increasing ampicillin concentration, which is distinct from the cell response to nalidixic acid as assessed by both methods.

### Recovery of *E. coli* cells after exposure to antibiotics

**Figure 3:**
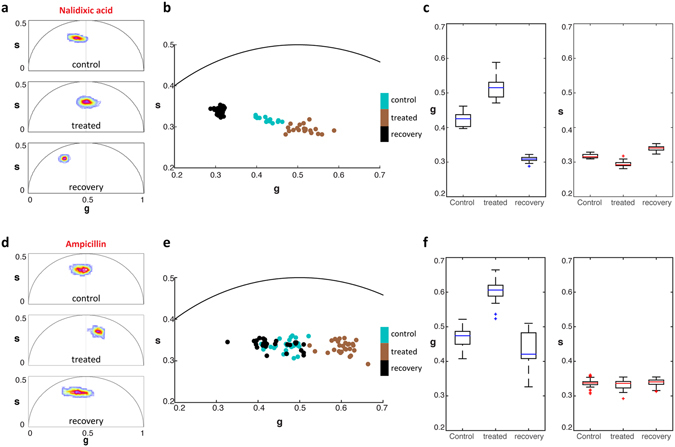
Bacterial phasor response to antibiotic treatment and recovery. (**a**) Phasor distributions of *E. coli* (control), *E. coli* treated with 0.464 mg/ml nalidixic acid for 30 min (treated), and treated *E. coli* recovered in fresh growth medium for 30 min (recovery). (**b**) Individual bacterial cell phasor of control, treated, and recovery groups exposed to nalidixic acid and their corresponding *g* and *s* distributions (**c**). (**d**) Phasor distributions of *E. coli* (control), *E. coli* treated with 0.4 mg/ml ampicillin for 30 min (treated) and treated *E. coli* recovered in fresh growth medium for 30 min (recovery). (**e**) Individual bacterial cell phasor of control, treated and recovery groups exposed to ampicillin and their corresponding *g* and *s* distributions (**f**).

To verify whether the bacterial phasor position correlates with global metabolic activity, *E. coli* cells were allowed to recover after exposure to both antibiotics. Cells were subjected to 0.464 mg/ml nalidixic acid for 30 min and FLIM data was acquired. The cells were then washed and incubated in growth media for 30 min and imaged again. As observed in the previous section, exposure to nalidixic acid shifted the phasor to the right, indicating a higher free to bound NAD(P)H ratio (Fig. 3a and b). When the cells recovered in media, the phasor distribution of bacteria shifted back towards the left, indicating a lower free to bound NAD(P)H ratio. The phasor of the recovered cells shifted to significantly longer lifetimes than the initial population before exposure to nalidixic acid. Further, the phasor distribution of individual cells within the population along the *g* axis was much narrower than both the initial control and antibiotic-exposed populations (Fig. 3a and c).

Ampicillin treated *E. coli* displayed similar shifts in cell phasors towards shorter lifetimes (Fig. 3d and e). However, recovery of these cells in fresh media showed a limited shift back to smaller *g* values compared to cells recovered from nalidixic acid exposure (Fig. 3c and f). The number of viable cells in the ampicillin treated samples was also lower by 1.8 times compared to the initial and nalidixic acid treated cells (Supplementary Fig. S6). The phasor distribution of cells in the recovered population was similar to the phasor of the cells in the initial population (Fig. 3f). In both cases, cells exposed to either antibiotic had phasors shifted more significantly along the *g* axis than along the *s* axis of the phasor plot in response to treatment (Fig. 3c and f). By comparing these phasor positions, changes in the ratio of free to bound NAD(P)H within *E. coli* cells were differentiated between initial and antibiotic-exposed cells. Further, the phasor positions and distribution of the recovered populations upon recovery from bacteriostatic and bactericidal antibiotics represent distinguishing metrics captured by FLIM.

### Bacterial phasors as a function of culturing time

**Figure 4:**
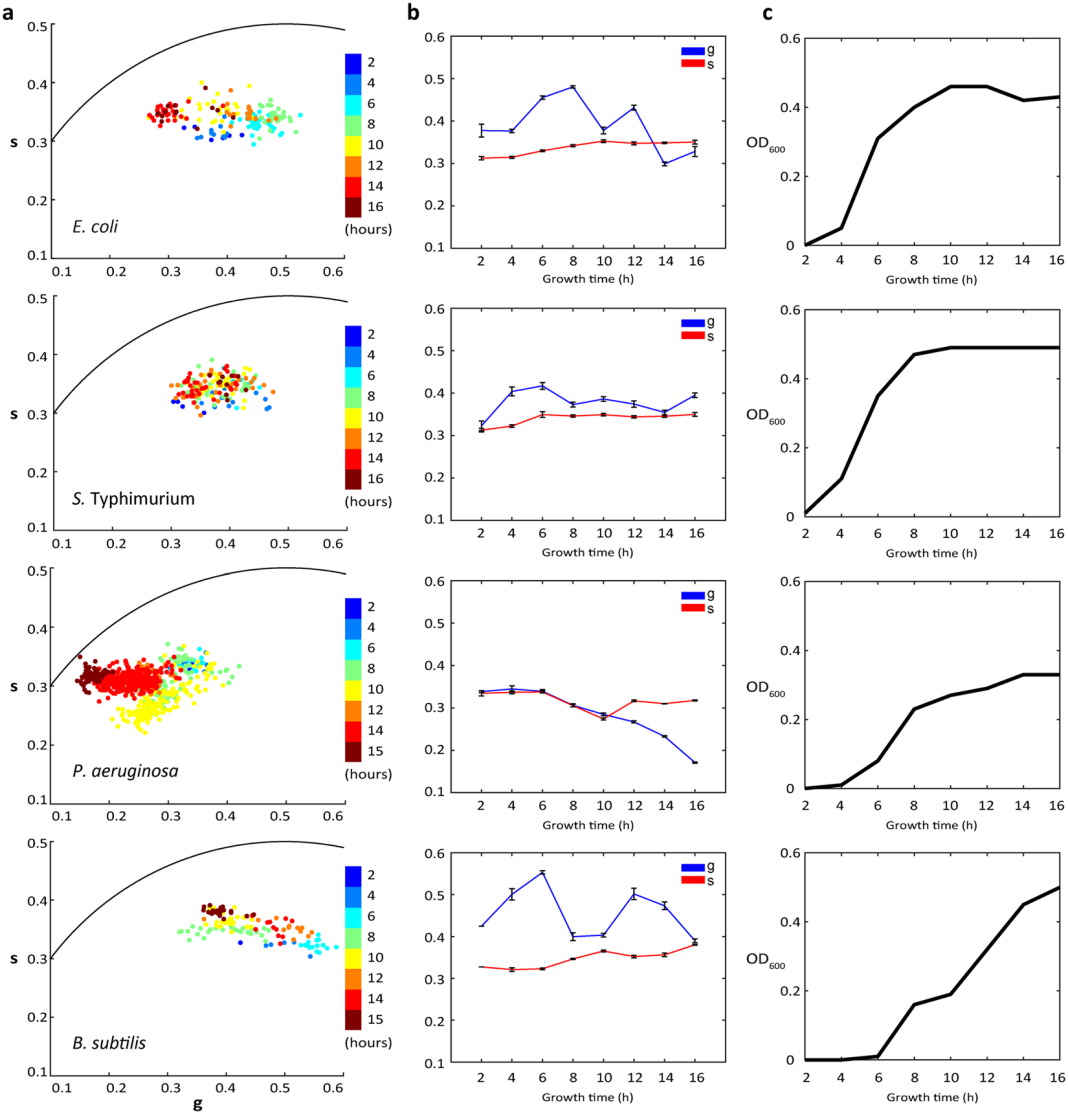
The effect of growth time on the bacterial phasor. (**a**) Bacterial phasor fingerprint of individual *E. coli, S*. Typhimurium*, P. aeruginosa*, and *B. subtilis* cells from aliquots taken at different shaking culture growth time points. (**b**) Mean *g* and *s* values along the growth curve, and (**c**) the corresponding optical densities of the shaking cultures.

To track the change in bacterial phasor position at different growth phases, we imaged *E. coli*, *S*. Typhimurium, *P. aeruginosa* and *B. subtilis* cells from shaking cultures at varied growth times (Fig. 4a and b). The cell density of these bacterial cultures was tracked in parallel using optical density (OD) measurements (Fig. 4c). FLIM data was collected from shaking culture aliquots at regular intervals, starting at 2 h growth time (Supplementary Fig. S7). No cells were found in the agarose-embedded samples at shorter times to acquire FLIM data. Comparing exponential phase cells, at 4 and 6 h, to the 2 h time point, all cultures except *P. aeruginosa* exhibited a shift in their mean phasor position to the right, i.e. a larger free to bound NAD(P)H ratio (Fig. 4a and b). At 8 h and beyond, the mean phasor position of *E. coli*, *S*. Typhimurium, and *B. subtilis* cells shifted back to smaller values of *g* and began to oscillate, to differing degrees, along the *g* axis as cultures reached the stationary phase (Fig. 4b). In *E. coli* and *B. subtilis*, these oscillations brought the mean phasor positions to even smaller *g* values than the cultures sampled at 2 h growth. *s* positions of the mean phasor were not as sensitive as the *g* position, but tended towards larger values, also non-monotonically, with culture time. As opposed to the other three species, the mean phasor of *P. aeruginosa* tended to smaller values in both *g* and *s*, and no oscillations were observed in *g* as a function of growth time.

### Growth recovery from extended stationary phase cultures

**Figure 5:**
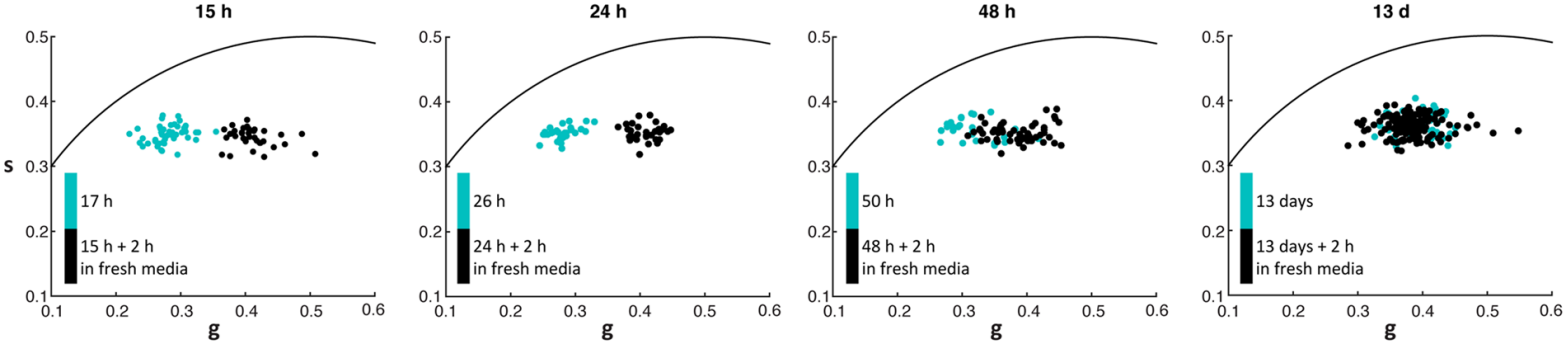
Bacterial phasor recovery after late stationary phase growth. Bacterial phasor of *E. coli* cells from 15 h, 24 h, 48 h and 13 d cultures recovered for an additional 2 h in spent medium from the same culture (cyan) or fresh growth medium (black).

The FLIM phasors of *E. coli* cells grown for 15 h, 24 h, 48 h and 307 h (13 days) were compared to those grown for the same amount of time and resuspended in fresh media. Figure 5 shows the cell phasor distribution of each of the growth time groups. We observed an increase in the ratio of free to bound NAD(P)H in the cells resuspended in fresh media when compared to their corresponding spent culture media counterparts at all time points except 13 day old cultures. The phasors of recovered cells remained at about the same position for each growth time point, but the phasors of cells taken directly from the spent culture medium shifted closer to the recovered cell phasor position with growth time. The difference in phasor position between the spent media and recovered cultures decreased with longer growth times. *E. coli* cells grown for 13 days did not show any change in their phasor position after 2 h recovery in fresh media.

## Discussion

In this work, we employ label-free NAD(P)H FLIM to characterize fluorescence lifetime fingerprint of bacteria with cellular resolution. The phasor approach to FLIM proves to be a powerful tool to differentiate metabolic states of bacteria via the relative quantification of free to bound NAD(P)H ratios. This ratio is expected to be related to metabolic activity, as NAD(P)+ reducing enzymes are central to catabolic pathways such as glycolysis, the citric acid cycle and the pentose phosphate pathway, which are each active in bacteria under different growth conditions. Similarly, enzymes such as NADH dehydrogenases and NAD(P)H oxidases play a central role in various anabolic pathways and show a high level of specificity in binding to NADPH or NADH^50–52^.

NAD(P)H FLIM of *E. coli, P. aeruginosa, B. subtilis, S*. Typhimurium and *S. epidermidis* was used to generate the bacterial species fingerprint phasors as well as those for the antibiotic experiments. We observed small variations between phasors of control populations of *E. coli* even when grown under the same conditions and harvested at the same time and OD, perhaps due to normal metabolic variability (Supplementary Fig. S8). The phasor fingerprint exhibits a distribution of values of individual cells within a population of same species (Fig. 1b and c). These differences suggest a distribution of metabolic activity within and between species populations at the same time of growth. The observed diversity of phasor positions may be a result of varying rates of NAD(P)+/NAD(P)H reduction/oxidation and shifts in NAD(P)H utilization due to catabolic and anabolic adaptations^32, 33, 53, 54^.

The shift of the NAD(P)H phasor position towards shorter lifetimes in response to antibiotic exposure suggests a decrease in enzyme binding of NAD(P)H, indicated by a larger free to bound ratio. In case of bacteriostatic antibiotics this shift tracks with lower respiration activity indicated by the resazurin assay (Supplementary Fig. S5). These results are consistent with previously reported data^46, 55^ which suggest that bacteriostatic antibiotics induce lower oxygen consumption rates (OCR) in cultures by arresting respiration in cells. The increase in respiration activity at low ampicillin concentrations, on the other hand, is consistent with findings that OCR increases upon exposure to bactericidal antibiotics^46, 55^. While the effects of higher concentrations of ampicillin on respiration have not been studied, to our knowledge, the resazurin and NAD(P)H FLIM data show a shift to a more reducing environment and an increase in the free to bound NAD(P)H ratio in the phasor plots, respectively.

In the event of cell death, the metabolic state is irreversible; however bacterial cells are known to recover from a state of static growth and dormancy induced by bacteriostatic compounds^9, 46, 47, 56^. The observed return of the mean phasor position to the left of the plot when nalidixic acid was washed away from the *E. coli* cells (Fig. 3a and b) is consistent with these previous results. In addition to the shift in the mean phasor, the distribution of single-cell phasors about the mean in the recovered population exposed to nalidixic acid was much narrower than the control and treated populations (Fig. 3c). In the context of the above results, the decrease in phasor variance indicates a recovery to a relatively homogeneous metabolic state throughout the population, as compared to the initial and nalidixic acid-treated populations, which exhibit a greater spread in NAD(P)H lifetimes.

In contrast to the recovery results from nalidixic acid, the shift of the phasor position of cells treated with ampicillin and recovered in fresh medium was less pronounced and their distribution was much broader (Fig. 3d). This phasor data was acquired from a population of cells that did not lyse as a result of the ampicillin treatment, so the extent of phasor recovery is of a biased population and characteristic only of cells that survived the treatment. Indeed, the density of cells that survived this treatment was 19.1 times lower than the nalidixic acid treatment (Supplementary Fig. S6). In addition, the phasor distribution of the recovered cell population was much broader than that of cells recovered from nalidixic acid treatment, indicating more metabolic heterogeneity. The response of bacteria to high-stress environments depends on a range of factors, such as the rate of cell division and initial metabolic states^9, 57–60^. Such factors can direct the fate of part of the population towards persister or dormant states and the rest towards death. Exposure to bactericidal antibiotics also results in generation of reactive oxygen species^61, 62^ and broad, and ultimately toxic, changes to the activity of central metabolic pathways in *E. coli* ^55, 62^. Consequently, the bactericidal effects of ampicillin, in contrast to nalidixic acid, may result in a population with the diversity of metabolic states observed in the recovered populations.

The metabolism of bacterial populations vary drastically over time, resulting in a collection of different metabolic states that cannot be captured by optical density measurements alone^63–65^. In the case of aerobically grown bacteria, these variations depend on the type of nutrient source, temperature of growth, and rate of aeration, among others. Moreover, the bacterial growth curve extends beyond the conventional lag, exponential and stationary phases of growth^66–68^. Many interesting physiological phenomena occur during late stationary phase growth, such as the emergence of persister cells and growth advantage in stationary phase (GASP) phenotypes, changes in gene expression and cell morphology, and programmed cell death^60, 68–71^. Most importantly each bacterium within the population may experience different metabolic and growth rates compared to neighboring cells, yet this information cannot be obtained by cell density measurement or CFU counts.

We employed NAD(P)H FLIM phasor to fingerprint the metabolic states of 4 bacterial species at different stages of growth (Fig. 4a). The earliest time point, 2 h, is already near the end of the lag phase for these cultures. The mean phasor positions of *E. coli*, *S*. Typhimurium, and *B. subtilis* all begin at relatively small *g* values. In comparison with observed phasors from the antibiotic exposure and recover experiments, these smaller *g* values suggest a greater metabolic activity. As cells enter their exponential phase, their phasors shift right to larger *g* values, consistent with a ramp down of metabolic rates as previously determined via indirect methods^64, 65^. At 8–10 h growth, this phasor trend reverses for these three cultures, suggesting an additional metabolic transition commensurate with the change in growth rate as cultures enter the stationary phase. In the stationary phase, their phasor positions shift back and forth in *g* in a manner seemingly unrelated to the cell density (Figs 4b and Supplementary S10). Bacterial cultures display an oscillatory behavior of cell density in the culture with rise and fall during the stationary phase due to GASP phenotypes^69, 71, 72^, but these typically occur at late stationary phase growth, and the observed phasor shifts are not directly correlated with cell density. Instead, these results indicate that the NAD(P)H phasor is sensitive to metabolic changes within the cells not captured by cell density or CFU analyses.

To look at metabolic adaptations in the stationary phase cultures, FLIM data was obtained from *E. coli* cultures grown for 15 h, 24 h, 48 h, and 13 d (Fig. 5). Interestingly, cells grown for an additional 2 h in the spent culture medium from 15, 24, and 48 h cultures had phasors at longer lifetimes than the early and mid-exponential phase cells in Figures 1, 2, 3 and 4 (*g* ~ 0.4). The NAD(P)H lifetimes in these populations were also longer than those of cells from those same cultures that recovered for an additional 2 h in fresh growth medium, which were themselves consistent with early exponential phase *E. coli* cells (Fig. 4), suggesting a growth recovery. The difference in mean phasor position between cells recovered in fresh and spent medium, however, shrinks with increasing culture time at 48 h and 13 d, indicating a diminished capacity of these bacteria to recover (Supplementary Fig. S11). Indeed, cells from the 13 d culture did not show any recovery, suggesting that most cells are dead or in a phenotypic state with an extended lag time to exponential growth. Future experiments will focus on FLIM and independent metabolic rate measurement in late stationary phase cultures to draw quantitative correlations between the two.

In conclusion, we have demonstrated a label-free, two-photon FLIM method for tracking changes in bacterial metabolism. Our results represent the first NAD(P)H FLIM phasor fingerprinting of various bacterial species, and they indicate that the NAD(P)H fluorescence lifetime, as captured in the phasor position, is sensitive to changes in metabolic states within cell populations. The NAD(P)H phasor of cells exposed to antibiotics exhibited the expected shifts due to diminished metabolic capacity, as well as detecting the recovery of cells resistant or tolerant to ampicillin exposure. The distribution of cell phasors within a population also changes as a function of treatment conditions, providing unique insight into single-cell and community behavior. Lastly, NAD(P)H FLIM of bacteria from planktonic cultures exhibited changes in the phasors that did not track with cell density, indicating that this technique can provide additional information about cell metabolism beyond what is inferred from conventional growth rate measurements. This work demonstrates the power of NAD(P)H FLIM to track metabolic states of individual bacteria *in situ*, and that the bacterial phasor represents a unique and complimentary set of data to conventional metabolism and growth characterization results.

## Material and Methods

### Bacterial strains and growth conditions

The bacterial strains used in this study are listed in Table S1. All strains were revived from frozen stocks by streaking on lysogeny broth (LB) agar (1.5%) plates. Shaking cultures of bacterial strains were grown in 2 ml LB at 37 °C for 5 h unless stated otherwise. For imaging, the samples were prepared by mixing LB shaking cultures with 1% agarose in a 3:7 ratio. 100 μl of the resulting solution was cast on a glass coverslip by spin coating at 500 rpm for 10 seconds.

### Exposure of agarose-embedded bacteria to antibiotics

Solutions of nalidixic acid or ampicillin (Sigma-Aldrich) were prepared at the indicated concentrations in sterile ultrapure water, well above the minimum inhibitory concentration in *E. coli*. The agarose-embedded bacterial samples were incubated with a 200 μl of each concentration for 30 min, then washed with sterile ultrapure water and imaged.

### Recovery of agarose-embedded bacteria from antibiotic exposure

Agarose-embedded bacterial samples on glass coverslip were incubated in 0.464 mg/ml nalidixic acid or 0.4 mg/ml ampicillin for 30 min. The samples were washed twice with ultrapure water, then incubated in LB media for 30 min and imaged.

### Measurement of cellular respiration

*E. coli* cells were grown shaking in LB media at 37 °C for 5 h. 300 µl of *E. coli* cells in LB media was diluted in 700 µl of PBS with different final concentrations of nalidixic acid and ampicillin. Each of these solutions were done in triplicates. The *E. coli* cells were incubated at 22 °C for 30 min. 20 µl of 0.15 mg/ml resazurin was injected into 100 µl of each of these solutions, and sterile control solutions, in a 96 well plate and the plates were incubated at 37 °C for 1 h. Fluorescence intensity from the wells was recorded using a plate reader (BioTek Synergy H1) with a 560 nm excitation/590 nm emission filter set.

### Growth curve sample preparation

For each growth curve, 20 ml LB media was inoculated with bacteria to an optical density at 600 nm (OD) of 0.02 and aliquoted into 1 ml cultures in 20 ml glass culture tubes with loose-fitting metal caps, which were kept shaking at 200 rpm and 37 °C. One culture aliquot was taken out for each bacterium every 2 h, and samples for FLIM measurements were prepared as described above.

The OD of 1 ml bacterial shaking cultures was measured every 2 h to generate a growth curve. The OD was measure at 600 nm using a Biowave CO8000 cell density meter (Biochrom, Holliston, MA).

### Growth curve recovery sample preparation

For growth curve recovery 1 ml cultures were grown for 15 h, 24 h, 48 h and 13 d in duplicates. 100 μl from one of the duplicate samples was diluted into 900 μl of fresh LB media and shaken for 2 h at 200 rpm and 37 °C. The other set of cultures from each time point was allowed to grow in the same spent culture media for 2 more hours. After 2 h both cultures were taken out of the incubator and samples were prepared for FLIM imaging as described above.

### FLIM data acquisition

FLIM was performed on a Zeiss LSM 710 microscope (Carl Zeiss, Jena, Germany) coupled to an 80 MHz multiphoton excitation laser source, Titanium:Sapphire MaiTai laser (Spectra-Physics, Mountain View, CA) with excitation at 740 nm using a 60X, 1.2 N.A. oil immersion objective, (Carl Zeiss, Oberkochen, Germany). The image scan speed was 25.21 µs/pixel with an image size of 256 × 256 pixels. Excitation from emission signal were separated at 690 nm followed by bandpass emission filter 460/80 nm (Semrock, Rochester, NY). Photomultiplier tube (H7422P-40, Hamamatsu, Japan) was used as the microscope external detector port photo-sensor unit. A320 FastFLIM FLIMbox (ISS, Champaign, IL) was employed to acquire frequency domain FLIM data. SimFCS software (LFD, Irvine) was used for FLIM data acquisition. For calibrating the FLIM system, Rhodamine 110 with known lifetime of 4 ns was measured for every experiment. The (*g*, *s*) coordinate system used to indicate phasor cursor coordinates in this article used the first harmonic phasor plots at 80 MHz (repetition rate of the laser).

### Spectral data acquisition

NAD(P)H in the bacterial samples was excited with two-photon excitation at 740 nm. Fluorescence spectra were collected on a Zeiss LSM 710 microscope spectral detector consisting of 32 channels, between 416–728 nm, each with 9.7 nm bandwidth. For image acquisition, the pixel frame size was set to 256 × 256 and the pixel dwell time was 177 μs/pixel. Spectral data were acquired from the same sample on which FLIM was performed.

### Data analysis

FLIM data was analyzed using the SimFCS software developed at the Laboratory for Fluorescence Dynamics (LFD, UC Irvine). To create the bacterial phasors, the average *g* and *s* value of phasor distribution from individual bacterial cell were calculated and plotted as a scatter plot. Thus, each point of the bacterial phasor scatter plot represents a single bacterium. Image segmentation, scatter plot and box plots calculations were performed on MATLAB. For Supplementary Fig. S2, the acquired fluorescence spectra were smoothed and normalized to maximum intensity.

### Live/dead assay

Agarose-embedded samples were exposed to different antibiotic concentrations for 30 min and stained with Syto 9 and propidium iodide for 20 min. The samples were then washed with ultrapure water and imaged using a Zeiss LSM780 inverted confocal microscope (Carl Zeiss, Jena, Germany) with 63X, NA 1.2 water immersion objective. Dichroic beam splitters were used to reflect laser lines at 488 and 561 nm. Both color channels were imaged using alternating line averaging to avoid blurring of the image due to drifting. These adjustments reduced fluorescence cross-talk to undetectable level in two-color imaging experiments. Confocal images were obtained with a pinhole size of 1 airy unit.

The fraction of dead cells was quantified using the “select objects” from the intensity images from each channel using the Volocity software package after deconvoluting the images by applying the point spread function to each channel.

### Colony forming unit (CFU) analysis

1 ml shaking cultures of E. coli were grown for 5 h and exposed to 0.464 mg/ml nalidixic acid or 0.4 mg/ml ampicillin for 30 min. The cells were centrifuged at 2000 × *g*, resuspended in LB media and incubated at 37 °C and shaking at 250 rpm for 30 min. The control was not exposed to antibiotics and each condition was done in triplicate. Serial dilutions of the shaking cultures were plated to count CFUs to measure recovery from antibiotic exposure.

## Electronic supplementary material

Supplementary Information
