# Supplementary material for: Metabolic fingerprinting of bacteria by fluorescence lifetime imaging microscopy
Source: Sci Rep. 2017 Jun 16;7:3743. doi: 10.1038/s41598-017-04032-w (PMC5473825; doi:10.1038/s41598-017-04032-w)
Supplement: Supplementary file 1 — Supplementary Information [file 41598_2017_4032_MOESM1_ESM.pdf]

Supplementary Information for:

Metabolic fingerprinting of bacteria by fluorescence lifetime imaging microscopy

Authors: *Arunima Bhattacharjee,[a†] Rupsa Datta,[b†] Enrico Gratton,[b] Allon I. Hochbaum [a,c*]*

Author affiliation: *[a] Department of Chemical Engineering and Materials Science, University of California, Irvine, Irvine, CA 92697, USA; [b] Laboratory for Fluorescence Dynamics, Department of Biomedical Engineering, University of California, Irvine, Irvine, CA 92697, USA; [c] Department of Chemistry, University of California, Irvine, Irvine, CA 92697, USA.*

Supplementary Information includes:

1. Supplementary figures, S1 – S11
2. Supplementary table, S1
3. Supplementary references

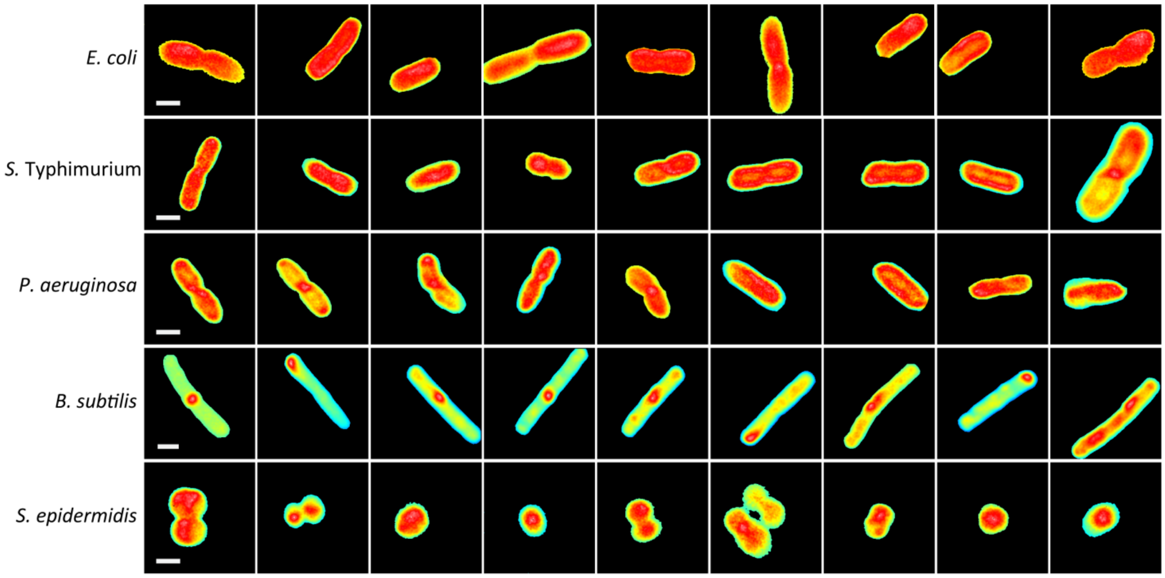

Supplementary Figure S1. Representative fluorescent intensity images of individual *E. coli, S.* Typhimurium*, P. aeruginosa*, *B. subtilis,* and *S. epidermidis* cells, excited with two-photon excitation at 740 nm. Scale bars are 1 µm.

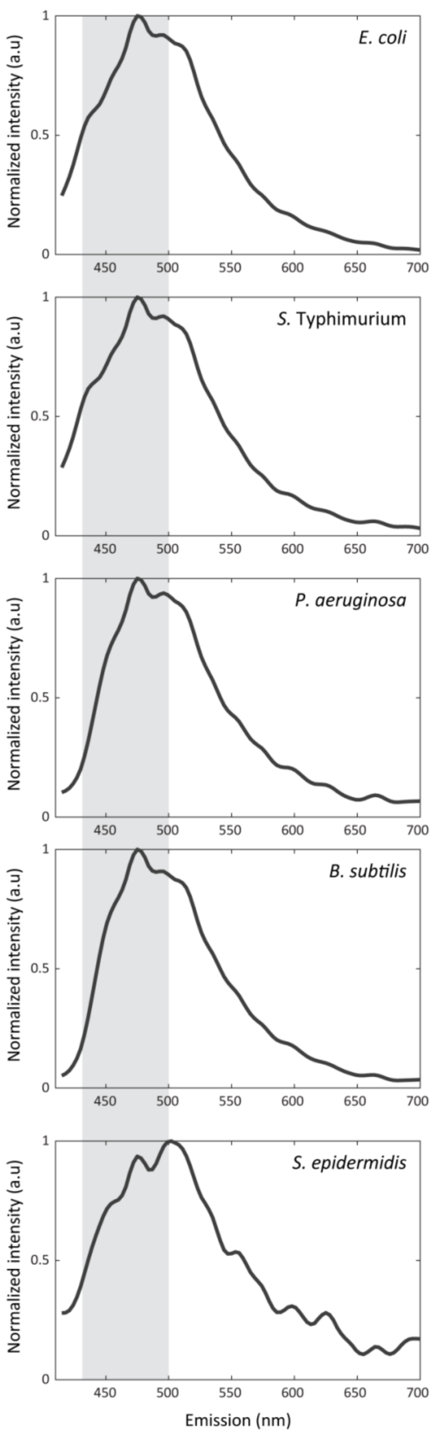

Supplementary Figure S2. Representative fluorescence emission spectra of *E. coli, S.* Typhimurium*, P. aeruginosa*, *B. subtilis,* and *S. epidermidis* cells, excited with two-photon excitation at 740nm. The grey band indicates the spectral region where FLIM data was collected.

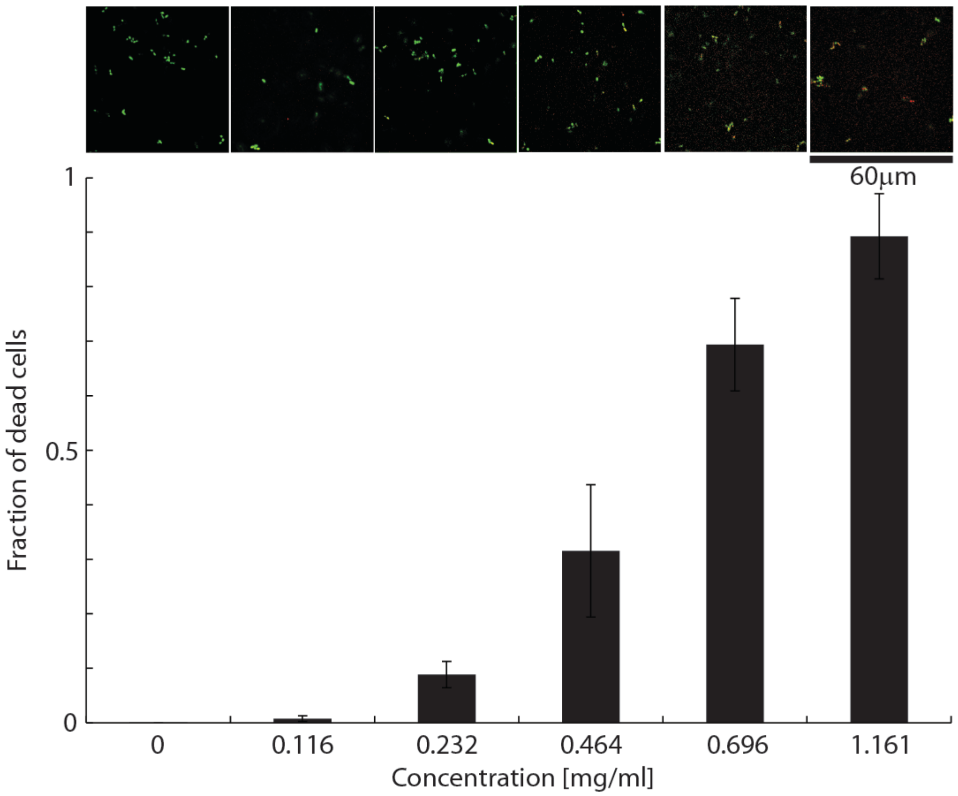

Supplementary Figure S3. Live/dead assay of cells exposed to nalidixic acid. Top panel shows representative images of *E. coli* cells stained with propidium iodide and syto 9 after being embedded in agarose and treated with different concentrations of nalidixic acid. Red and green color represent dead and live cells respectively. Lower panel shows the fraction of dead cells in each sample from image analysis of live/dead staining.

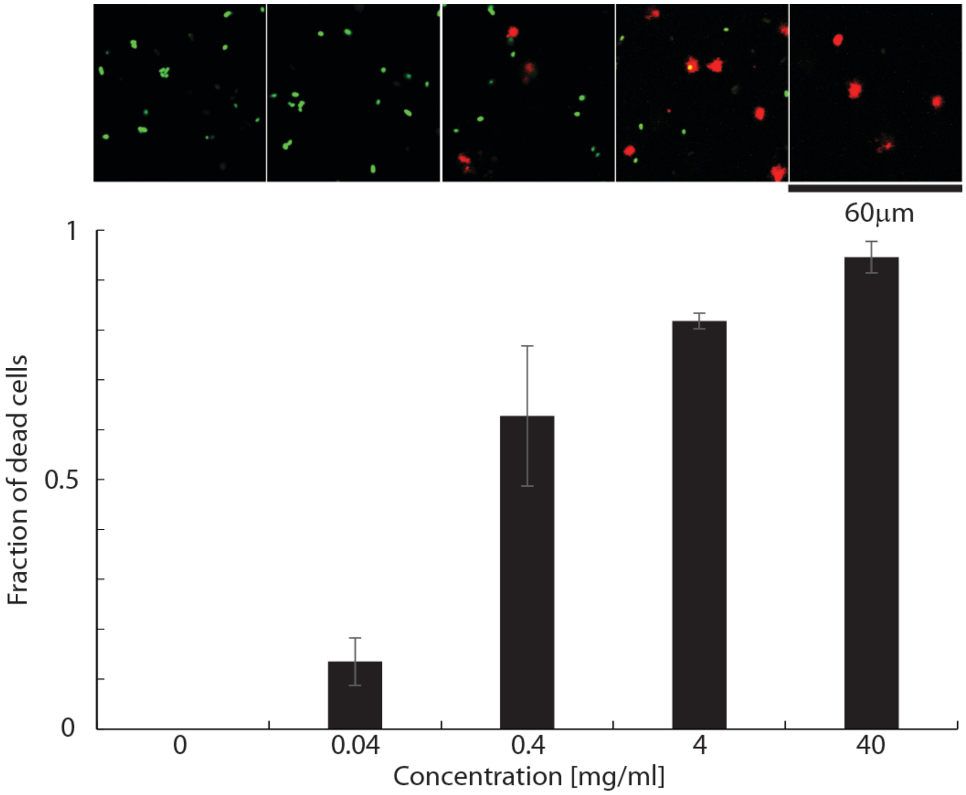

Supplementary Figure S4. Live/dead assay of cells exposed to ampicillin. Top panel shows representative images of *E. coli* cells stained with propidium iodide and syto 9 after being embedded in agarose and treated with different concentrations of ampicillin. Red and green color represent dead and live cells respectively. Lower panel shows the fraction of dead cells in each sample from image analysis of live/dead staining.

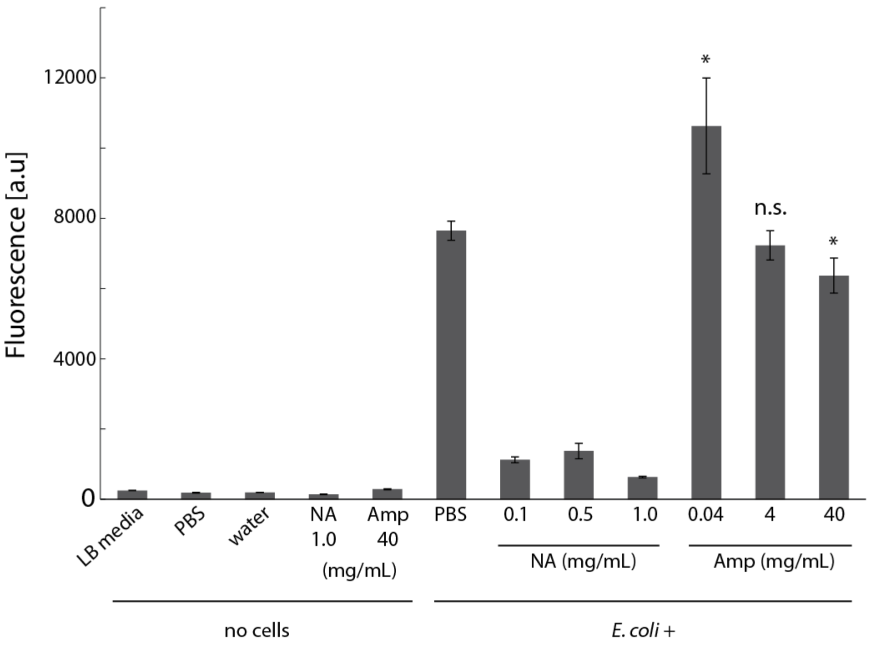

Supplementary Figure S5. Resazurin assay for measuring respiration in *E. coli* cells exposed to antibiotics. *E. coli* cells were exposed to different concentration of nalidixic acid (NA) and ampicillin (Amp) and for 30 min before injecting with resazurin to measure respiration. The fluorescence of resazurin control solutions without *E. coli* cells show low fluorescence, indicative of non-existent metabolic activity, while the control sample, *E. coli* cells exposed to PBS, shows increased fluorescence. * = $p < 0.05$ for comparisons of Amp treated cells to the *E. coli* + PBS control.

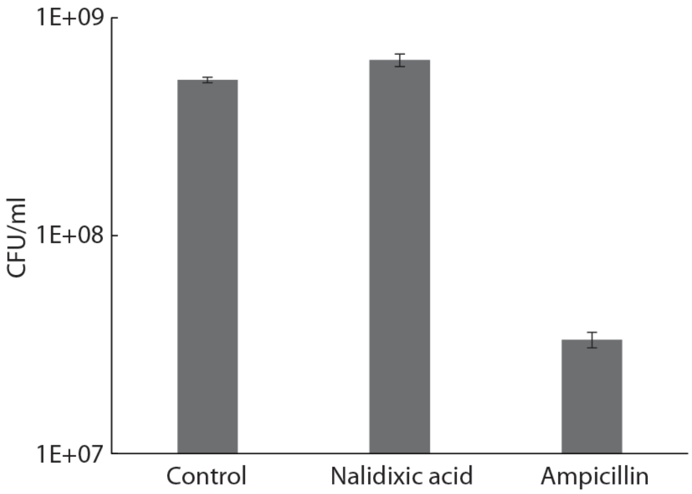

Supplementary Figure S6. Viability (CFU/ml) of *E. coli* cells plated on LB agar plated after exposure to antibiotics, nalidixic acid and ampicillin, and recovered in LB media. The control is not exposed to antibiotics.

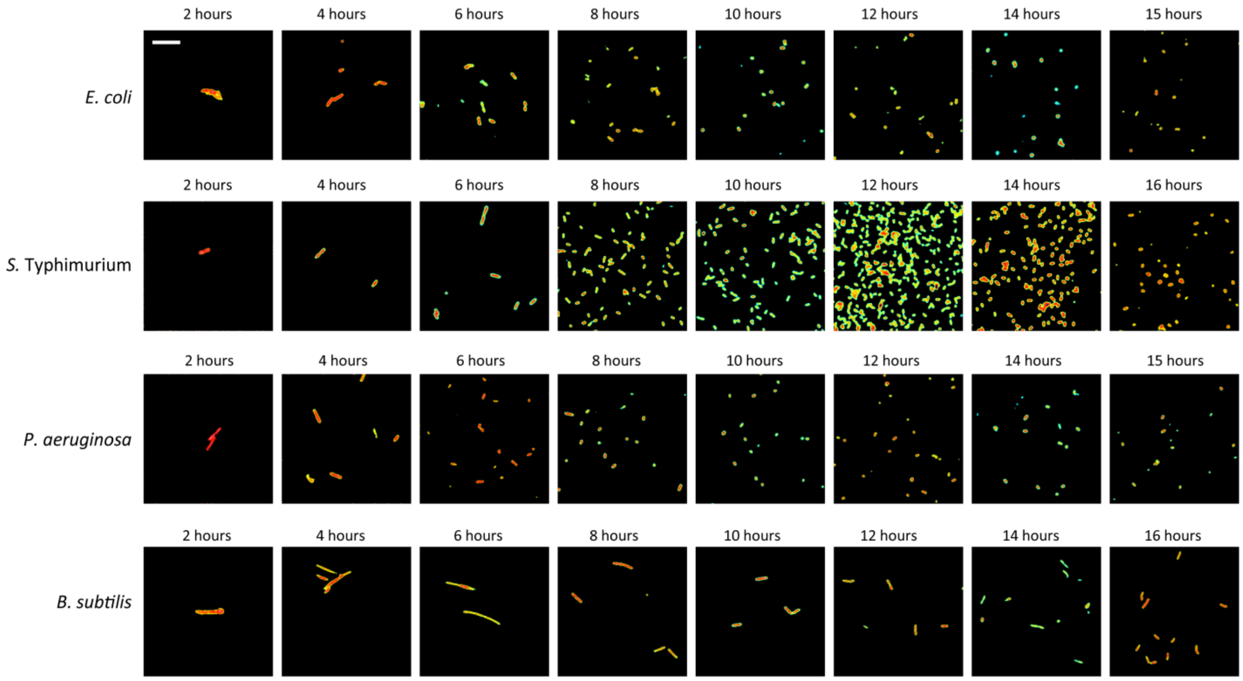

Supplementary Figure S7. Representative fluorescence intensity images of *E. coli, S.* Typhimurium*, P. aeruginosa*, *B. subtilis,* and *S. epidermidis* cells at different stages of planktonic growth. All images were obtained at the same magnification, and the scale bar is 10 μm

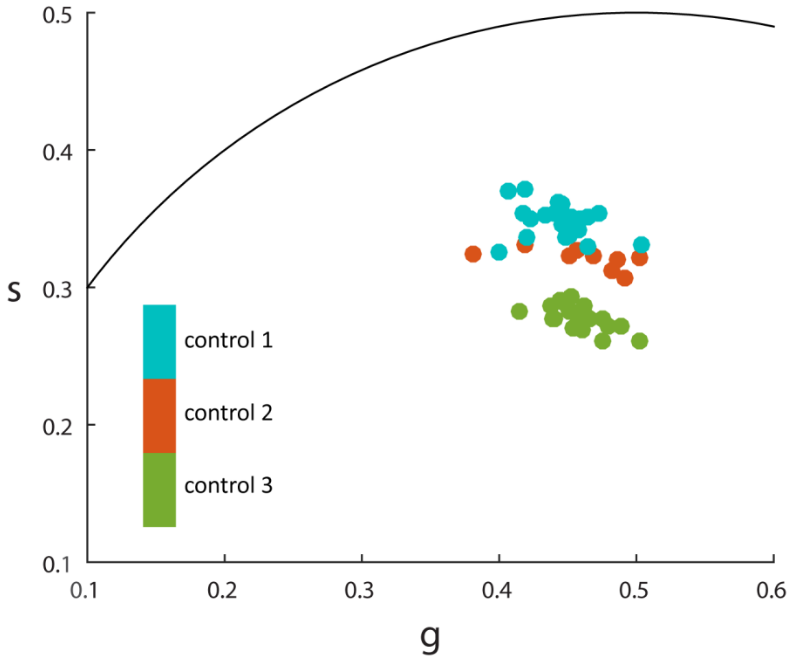

Supplementary Figure S8. Bacterial phasors of *E. coli* cells from three independent 5 h cultures grown on different days.

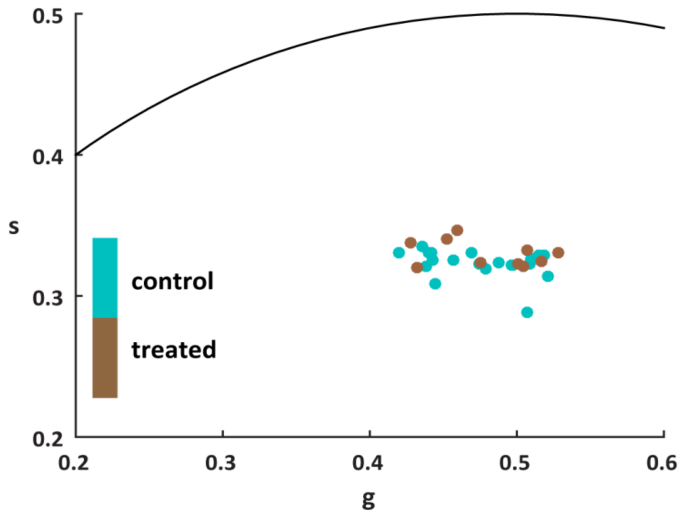

Supplementary Figure S9. Bacterial phasor of 5 h *E. coli* (control) culture and 5 h *E. coli* culture washed and incubated in water for 30 mins (treated).

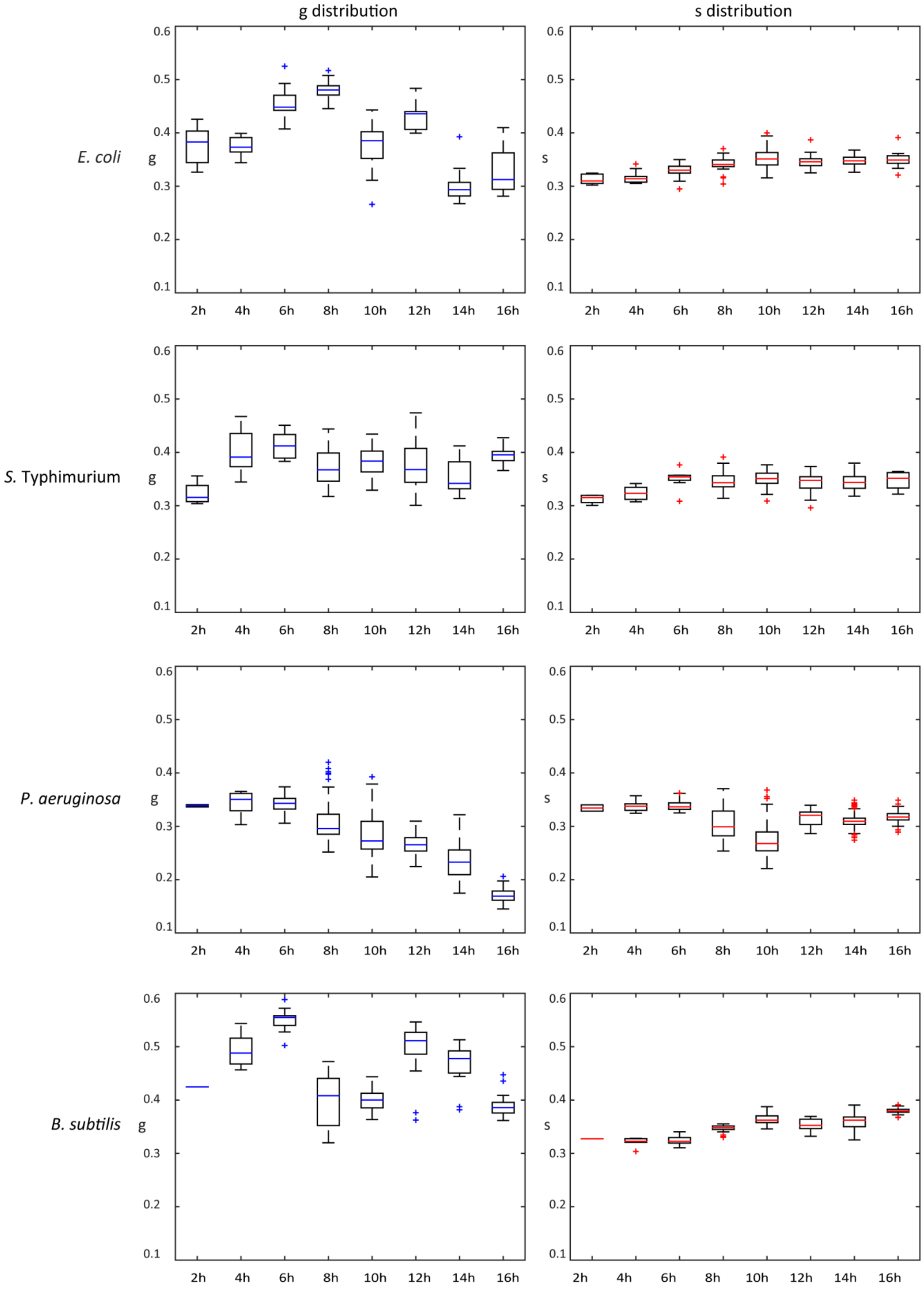

Supplementary Figure S10. *g* and *s* distributions of *E. coli, S.* Typhimurium*, P. aeruginosa*, *B. subtilis,* and *S. epidermidis* populations at different time points on the growth curve.

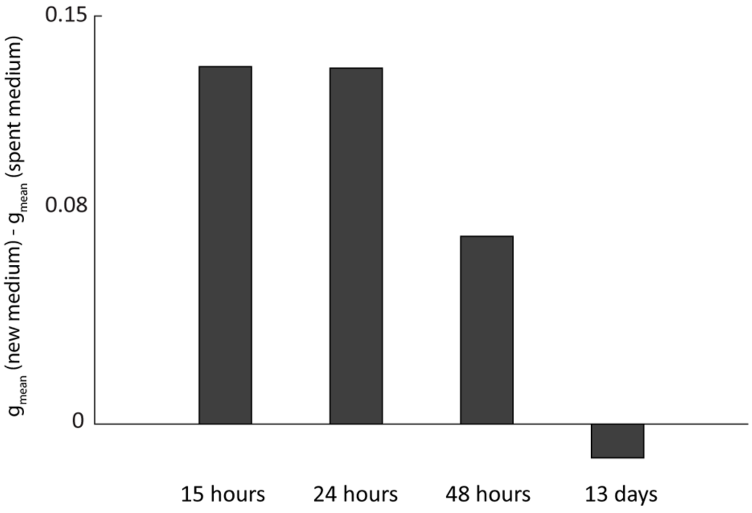

Supplementary Figure S11. Differences between mean *g* (g_{mean}) of the bacterial phasor distributions of 15 h, 24 h, 48 h, and 13 d *E. coli* cultures incubated in fresh media for 2 h compared to those incubated for a further 2 h in their spent media.

Supplementary Table S1

Bacterial strains		
Strain	Relevant properties	Source
E. coli K12 MC4100	*Escherichia coli* K12 derivative. *araD* Δ*(argF-lac)U169 rpsL relA flbB deoC ptsF rbsR*	[1]
P. aeruginosa PA14	*Pseudomonas aeruginosa* wild type clinical isolate	[2]
S. Typhimurium	*Salmonella enterica* serovar Typhimurium wild type isolate	ATCC 14028
B. subtillis	*Bacilus subtillis* wild type isolate	[3]
S. epidermidis	*Staphylococcus epidermidis*	ATCC 14990

Supplementary references

1. Casadaban, M. J. Transposition and fusion of the lac genes to selected promoters in Escherichia coli using bacteriophage lambda and Mu. *J. Mol. Biol.* **104,** 541–555 (1976).
2. Rahme, L. G. *et al.* Common virulence factors for bacterial pathogenicity in plants and animals. *Science* **268,** 1899–1902 (1995).
3. Branda, S. S., González-Pastor, J. E., Ben-Yehuda, S., Losick, R. & Kolter, R. Fruiting body formation by Bacillus subtilis. *Proc. Natl. Acad. Sci. U. S. A.* **98,** 11621–11626 (2001).
